# The Relationship Between External Load and Player Performance in Elite Female 3 × 3 Basketball Games: A Markerless Motion Capture Approach

**DOI:** 10.3390/s25206334

**Published:** 2025-10-14

**Authors:** Mingjia Qiu, Rui Dong, Junye Tao, Zhaoyu Li, Wen Zheng, Mingxin Zhang

**Affiliations:** 1 School of Athletic Performance, Shanghai University of Sport, Shanghai 200438, China; 2121151084@sus.edu.cn (M.Q.); 2321852032@sus.edu.cn (W.Z.); 2 Shanghai Key Lab of Human Performance, Shanghai University of Sport, Shanghai 200438, China; 3 School of Physical Education and Sports Science, South China Normal University, Guangzhou 510631, China; dongrui@scnu.edu.cn; 4 College of Physical Education and Sports, Beijing Normal University, Beijing 100875, China; 202411070032@mail.bnu.edu.cn; 5 Institute of Physical Education and Training, Capital University of Physical Education and Sports, Beijing 100091, China; lizhaoyu2022@cupes.edu.cn

**Keywords:** team sports, 3 on 3 basketball, physical demands, player monitoring

## Abstract

**Background**: This study employed a markerless motion capture system to quantify the external game load of elite 3 × 3 basketball players and evaluated its association with game performance. **Methods**: Twenty-four female 3 × 3 basketball games from the 2024 Paris Olympic Games were analyzed, involving 32 players from eight national teams. A markerless motion capture system was used to collect six categories of external load metrics during games, and 22 types of technical statistics were gathered to determine performance. Collected data were standardized according to live game time (min^−1^). Repeated-measures correlation analysis was applied to examine the relationships between external load and performance, while mixed-effects models were used to compare external load differences between better- and worse-performing groups (classified by Player Value). **Results**: The correlations between external load and performance indicators were trivial to small. Accelerations (ACC) were significantly associated with the greatest number of performance indicators (e.g., points, rebounds, 1-point made, key assists), while rebounds were significantly correlated with the largest number of external load metrics (e.g., total distance, low-intensity active distance, high-intensity active distance); however, all correlations remained at the small level (r = 0.16–0.24). No significant differences in external load were observed between players of differing performance groups (*p* > 0.05). **Conclusions**: In elite 3 × 3 basketball, external load reflects players’ involvement and effort rather than serving as a primary determinant of game performance. This study provides new empirical evidence on the characteristics of 3 × 3 basketball, suggesting that coaches and strength and conditioning practitioners should adopt a comprehensive perspective when evaluating performance, with external load being more suitable for training regulation and fatigue monitoring.

## 1. Introduction

In April 2025, the International Olympic Committee announced that the number of participating teams in 3 × 3 basketball would be expanded from 8 to 12 per gender at the upcoming 2028 Los Angeles Olympic Games. This initiative not only provides more nations with the opportunity to compete on the Olympic stage, but also further consolidates 3 × 3 basketball’s status as a flagship project promoted by the International Basketball Federation (FIBA). The rules of 3 × 3 basketball (10-min games, 12-s shot clock), along with simplified court dimensions and reduced player numbers, accelerate game tempo and increase the frequency of transitions, making it a high-intensity intermittent sport [1,2]. During games, players perform numerous accelerations, decelerations, changes in direction, jumps, and physical contests. On average, 3 × 3 basketball involves ~24% of playing time at high intensity, compared with ~9% in traditional 5 × 5. In terms of event density, players in 3 × 3 execute approximately 1.1–2.1 accelerations·min^−1^, 1.5–2.8 decelerations·min^−1^, and 3.9–5.9 changes in direction·min^−1^, which is consistently higher than the corresponding 0.6–0.8, 1.1–1.7, and 3.6–4.5·min^−1^ observed in 5 × 5 basketball [1]. This dense physical output pattern results in heart rates consistently exceeding 90% of maximum [3], accompanied by a rapid increase in blood lactate concentration. On average, blood lactate values in 3 × 3 basketball (~6.1–6.3 mmol·L^−1^) are broadly comparable to those in 5 × 5 basketball (≈6.5  ±  2.1 mmol·L^−1^ in females; up to ≈6.8  ±  2.8 mmol·L^−1^ in males) [4,5]. It should be noted that 3 × 3 games are of much shorter duration than 5 × 5, and that lactate values are influenced by the timing of sample collection. These studies reflect the high intensity reached during competition in this sport and its heavy reliance on anaerobic metabolic capacity. Moreover, the scheduling of 3 × 3 basketball is often organized as short-term tournaments, where players frequently play multiple games within a single day and are required to perform high-intensity tasks under conditions of incomplete recovery [6]. In the absence of scientific load monitoring, players are prone to fatigue or overload, which may impair tactical execution and competitive performance. Therefore, establishing a scientific framework for load monitoring and performance evaluation is crucial for this emerging Olympic discipline. However, research on the characteristics and demands of 3 × 3 basketball has remained limited over the past decade, highlighting the urgent need for empirical studies based on real-game contexts to better inform researchers and practitioners.

External load generally refers to the objective physical stimuli experienced by players during competition or training [7,8]. The monitoring and quantification of external load have been widely applied in team sports, providing important references for training regulation and performance evaluation. By tracking external load, coaches can ensure that players receive appropriate stimuli, thereby reducing the risks of non-functional overtraining, injuries, and illness [9,10]. With technological advancements, researchers can objectively measure players’ physical workload at both macro levels (e.g., PlayerLoad, running distance) and micro levels (e.g., acceleration changes, directional shifts). Traditionally, inertial measurement units (IMUs), local positioning systems (LPS), and integrated microsensor devices (e.g., Catapult) have been the primary tools, and their validity has been demonstrated in basketball training and certain official games [11,12]. Similarly, the application of these technologies has provided valuable tools for researchers and practitioners of 3 × 3 basketball to further understand the characteristics of the sport [4,5]. However, the application of traditional load-monitoring technologies in official 3 × 3 basketball games faces environmental limitations and regulatory constraints.

In official 3 × 3 basketball games, due to complex venue environments and restrictions at major international competitions (e.g., the Olympic Games) prohibiting players from wearing sensors, external load data can only be obtained through time-consuming and inefficient time-motion analysis [13,14]. In contrast, advances in computer vision and artificial intelligence over the past decade have driven the emergence of markerless motion capture systems. These systems do not require players to wear devices; instead, they rely on multi-view video and algorithms for human keypoint detection and 3D pose reconstruction, and they have been applied in the sports domain [15,16]. Initially, this approach was primarily used in sports biomechanics laboratories to support precise motion capture, posture estimation, and gait analysis in clinical or laboratory settings, and multiple studies have confirmed its good agreement with traditional systems such as IMUs and Vicon [17,18,19,20]. Subsequently, this approach was extended to performance analysis. Researchers incorporated it into training and real-game contexts, and it has been applied in sports such as badminton and tennis [21,22]. In these studies, researchers extracted valid external load data using markerless motion capture systems. With advantages such as deployment flexibility, efficiency, and non-invasiveness, markerless motion capture systems are gradually becoming a feasible alternative for load monitoring in real-game environments of team sports.

In basketball research, both external and internal load measures have been examined in relation to player performance [23,24,25,26]. External load is commonly quantified using indicators such as PlayerLoad, total distance covered, accelerations/decelerations, and actions across different intensity zones, while internal load is typically assessed through session rating of perceived exertion (sRPE). Player performance has generally been evaluated using box-score statistics, efficiency ratings, or performance index rating (PIR). Evidence from 5 × 5 basketball suggests that external load during official games shows small-to-moderate associations with performance, varying across competition level, age, and sex [23,24,25]. Vázquez-Guerrero [26] further applied cluster analysis to classify performance levels (better, intermediate, worse) and reported no significant differences in training load across these groups. Regarding internal load, studies indicate moderate associations with performance outcomes [25]. However, this body of evidence is derived primarily from 5 × 5 basketball. Although recent research has demonstrated strong correlations between external load (Movement Load) and internal load (sRPE, summated-heart-rate-zones) in 3 × 3 basketball, to our knowledge no study has yet systematically examined the relationship between external load and performance in 3 × 3 players.

Considering the differences between 3 × 3 and 5 × 5 basketball in game rules and characteristics, as well as in technical–tactical, physiological, physical, perceptual, and competition scheduling demands [1], findings from 5 × 5 cannot be directly generalized to 3 × 3, as such structural and demand-related differences may alter load–performance relationships and physiological responses across formats. In fact, existing studies have already highlighted the particular significance of external load in 3 × 3 basketball. Sansone and colleagues [2] recorded the “Movement Load” of adult male players across multiple official games and examined its correlations with heart rate load and ratings of perceived exertion (RPE). They found strong positive correlations with both physiological and subjective responses (r = 0.57–0.76), revealing the important feedback role of external load in monitoring players’ status in 3 × 3 basketball; however, its relationship with game performance was not further tested. Particularly given existing evidence suggesting that sex influences players’ physical output during competition [13], targeted studies on female samples have long been neglected. Such information is crucial for 3 × 3 basketball practitioners, guiding them in selecting workload thresholds in practice and providing gender-sensitive data to inform training optimization and game management.

Therefore, the purpose of this study was to quantify players’ external load using a markerless motion capture system in real 3 × 3 basketball games and to systematically examine its relationship with game performance. Specifically, this study (1) analyzed the correlations between external load indicators (e.g., PlayerLoad, running distance, number of accelerations) and performance metrics; and (2) classified players’ performance into high and average groups based on overall performance levels, and compared the external load differences between groups. We hypothesized that external load indicators would show significant associations with game performance metrics, but that the magnitude of these relationships would be small-to-moderate, reflecting the complex and multifactorial nature of basketball performance. Additionally, we expected that players with better performance would show higher external loads than those with worse performance.

## 2. Materials and Methods

### 2.1. Participants and Design

Twenty-four group-stage games from the female 3 × 3 basketball tournament at the 2024 Paris Olympic Games were monitored, covering all players from eight national teams (*n* = 32; age = 29.4 ± 4.7 years; height = 182.6 ± 7.4 cm). This study involved no intervention, as players competed in publicly accessible game settings. Strict anonymization procedures were applied during data processing to ensure that no personally identifiable information of players was included. The study strictly adhered to the principles of the Declaration of Helsinki, complied with ethical guidelines for sport and exercise science, and was approved by the Ethics Committee of Shanghai University of Sport [approval number: 102772025RT096].

This study employed a descriptive, observational, and retrospective design to explore the relationship between external load and players’ game performance. External load data were extracted using a markerless motion capture system, while performance data were obtained from the official statistics provided on the FIBA website.

### 2.2. Data Collection

**Figure 1:**
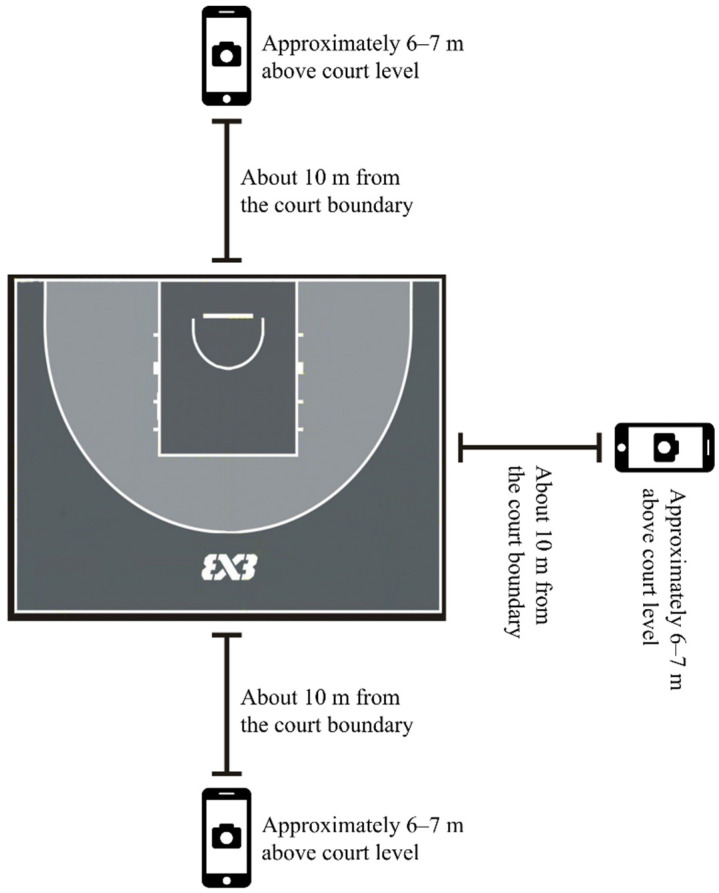
Schematic diagram of video recording setup.

To quantify external load during competition, this study employed a video-based markerless motion capture method. Researchers recorded multi-angle video of the 3 × 3 basketball games at the Paris Olympics: three Apple iPhone 13 devices (iOS 18, Apple Inc., Cupertino, CA, USA) were mounted at elevated positions in the front and side stands, approximately 6–7 m above court level and about 10 m from the court boundary, ensuring full-court coverage. Each device recorded in 1080p at 25 fps, with all other camera parameters maintained at default settings. A schematic of the setup is presented in Figure 1 to illustrate the measurement environment and ensure reproducibility. The recorded videos were subsequently edited following established procedures in basketball time–motion analysis, retaining only periods when the game clock was running (i.e., live play), and excluding stoppages due to fouls, timeouts, free throws, or out-of-bounds situations [27,28]. Video processing and analysis were conducted by an experienced basketball video analyst, with over three years of professional club experience, using SportsCode Elite software (v12, Hudl, Australia). For reliability assessment, the analyst reprocessed the game videos, yielding excellent intra-observer consistency (ICC = 0.92). Player performance data were collected from the official FIBA website, with indicators defined by the FIBA 3 × 3 statistics manual and previously applied in 3 × 3 basketball research [29], as shown in Table 1.

### 2.3. Markerless Motion Capture System

The external load data in this study were obtained using a markerless motion capture system based on Real-Time Models for Pose estimation (RTMPose) technology. RTMPose is a rapidly developing real-time pose estimation technique that can simultaneously identify skeletal keypoints of multiple players and track their dynamic movement trajectories. This method detects 17 key body points in video, aligned with pose detection from the authoritative Microsoft Common Objects in Context (MS COCO) dataset [30], including joints or endpoints of the head, trunk, and limbs [31]. In frame-by-frame computation, the two-dimensional coordinates of keypoints are first extracted, and then the three-dimensional coordinate system is reconstructed using triangulation under multi-view geometric constraints [32], thereby obtaining the complete skeletal information of players in the scene. Players’ external load was calculated based on the trajectory changes of these keypoints. Previous studies have demonstrated that RTMPose performs well on public datasets, achieving a mean accuracy of 75.8% on the MS COCO validation set [31,33]. The technique has been validated in applied studies on gait and human kinematics [34,35,36], with reliability and accuracy comparable to traditional methods such as inertial measurement units (IMUs) [18]. The RTMPose technology was implemented using Python 3.6 (Python Software Foundation, version 3.6). The detailed code implementation of RTMPose can be found on GitHub (https://github.com/open-mmlab/mmpose/tree/1.x/projects/rtmpose (accessed on 10 March 2024)).

The validity and reliability of the RTMPose-based markerless motion capture system used in this study were supported by internal comparison tests and external published evidence. We conducted comparison tests with the widely recognized Vicon marker-based motion capture system, which is considered the gold standard in biomechanical analysis. During the execution of typical basketball movement tasks by participants, we employed a custom marker set (ankle, knee, hip, shoulder, elbow, and wrist) rather than standardized models such as Plug-in-Gait or Helen Hayes, which are primarily designed for gait analysis and full-body kinematics/kinetics. This configuration focused on the joint trajectories most relevant to basketball-specific tasks, avoiding redundant inverse-dynamics modeling that was not aligned with our study aims. Simultaneous data collection revealed high spatial agreement between the two systems, with a mean intra-class correlation coefficient (ICC) of 0.89 (range: 0.77–0.97), indicating strong consistency in joint displacement trajectories. These results are consistent with recent validation work [37], which evaluated the RTMPose-based markerless motion capture system during standardized 3 × 3 basketball tasks. Their study reported that, compared with tape-measure benchmarks, displacement estimation bias and standard error of estimate (SEE) were below 5%, and velocity estimation showed an ICC range of 0.97 to 1.00 compared with manual time–motion analysis. In addition, recent work [38] further demonstrated the robustness of this system under complex game conditions, showing that it could reliably capture 3D trajectories of the head, trunk, and limbs even during high-speed movements, body occlusions, or overlapping trajectories of multiple players. Overall, the RTMPose-based markerless motion capture system provides an efficient, reliable, and ecologically valid tool for extracting external load in real basketball game environments.

### 2.4. External Load Metrics

Based on indicators previously used in traditional basketball and 3 × 3 basketball studies [4,5,11,39,40,41], six categories of external game load metrics were collected, as follows: (1) PlayerLoad, a reliable measure of external load, defined as the instantaneous rate of change in acceleration along the transverse (x), coronal (y), and sagittal (z) axes. This is calculated as the square root of the sum of squared changes in acceleration across the x, y, and z planes, multiplied by a scaling factor of 0.01. (2) Total distance, referring to the distance covered by players during the game. (3) Low-intensity, (4) moderate-intensity, and (5) high-intensity active distances (LIA, MIA HIA), representing movements distinct from standing, walking, or jogging, performed at low intensity (<2.5 m/s), moderate intensity (2.5–3.5 m/s), and high intensity (>3.5 m/s), respectively. (6) Accelerations (ACC): high-intensity forward or backward movements characterized by effort and intent approaching maximal levels (>3.5 m/s). All metrics were normalized to game time (min^−1^) to account for variations in game duration and playing time. Intra-class correlation coefficient (ICC) calculations for repeated measures of external load indicators showed excellent consistency (ICC = 0.98).

### 2.5. Statistical Analysis

Multiple repeated-measures correlation analyses were conducted between external load metrics and game performance to evaluate their relationships. Because players were monitored across multiple games, this approach explicitly accounted for repeated measures within players. The method was implemented using the rmcorr package in RStudio (version 4.1). According to Hopkins’ definitions [42], correlation coefficients (r) were interpreted as follows: trivial (r < 0.1), small (0.1 < r < 0.3), moderate (0.3 < r < 0.5), large (0.5 < r < 0.7), very large (0.7 < r < 0.9), almost perfect (r > 0.9), or perfect (r = 1).

To determine whether external load could differentiate player performance, k-means cluster analysis was applied to classify each player’s game Player Value (P-VAL) into better performance (*n* = 63, P-VAL = 8.58 ± 2.67) and worse performance (*n* = 128, P-VAL = 2.82 ± 1.81). This method is commonly used in performance analysis research as an unsupervised method of dividing data into two categories [43,44,45]. Independent linear mixed-effects models were then constructed, accounting for repeated measures within individuals. Specifically, each external game load metric was modeled as the dependent variable, with player performance as a fixed effect (two levels: better vs. worse performance) and players as a random effect (random intercept and fixed slope). *p*-values were adjusted using Bonferroni post hoc tests. Estimated marginal means and their 95% confidence intervals were calculated for each external load metric as descriptive statistics. Statistical significance was set at *p* < 0.05. All statistical analyses were performed using IBM SPSS software (version 24; IBM Corp, Armonk, NY, USA).

## 3. Results

**Figure 2:**
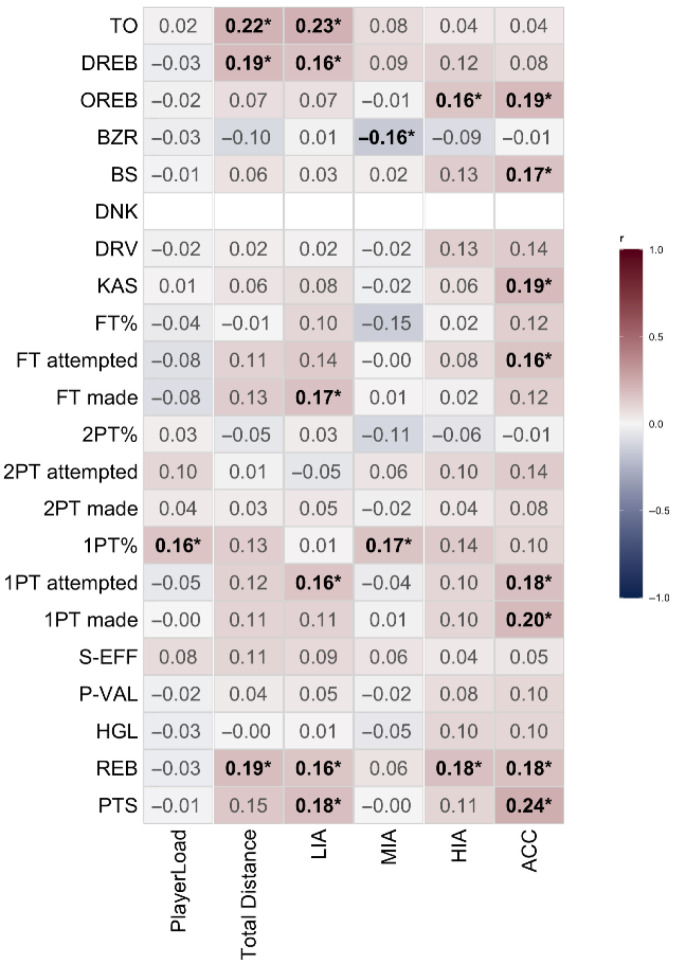
Heatmap of the correlation between external load and player performance. * Indicate ***p*** < 0.05.

The correlations between players’ external loads and game performances are presented in Figure 2. PlayerLoad showed a small positive correlation with 1PT% (r = 0.16, *p* = 0.042). Total Distance demonstrated small positive correlations with REB (r = 0.19, *p* = 0.018), DREB (r = 0.19, *p* = 0.014), and TO (r = 0.22, *p* = 0.005). LIA showed small positive correlations with PTS (r = 0.18, *p* = 0.021), REB (r = 0.16, *p* = 0.043), 1PT attempted (r = 0.16, *p* = 0.042), FT made (r = 0.17, *p* = 0.031), DREB (r = 0.16, *p* = 0.038), and TO (r = 0.23, *p* = 0.004). MIA showed a small positive correlation with 1PT% (r = 0.17, *p* = 0.034) and a small negative correlation with BZR (r = −0.16, *p* = 0.040). HIA showed small positive correlations with REB (r = 0.18, *p* = 0.023) and OREB (r = 0.16, *p* = 0.044). ACC showed small positive correlations with PTS (r = 0.24, *p* = 0.003), REB (r = 0.18, *p* = 0.026), 1PT made (r = 0.20, *p* = 0.013), 1PT attempted (r = 0.18, *p* = 0.013), FT attempted (r = 0.16, *p* = 0.040), KAS (r = 0.19, *p* = 0.014), BS (r = 0.17, *p* = 0.033), and OREB (r = 0.19, *p* = 0.014).

Comparisons of external load between players with better and worse performance are presented in Table 2. No significant differences were found in any external load indicators (*p* > 0.05).

## 4. Discussion

The purpose of this study was to evaluate the relationship between external load and game performance in female 3 × 3 basketball players during the Olympic Games, and to determine whether external load can distinguish between players’ performance levels. The results showed that, in elite-level female 3 × 3 basketball, correlations between external load indicators and most game performance metrics were trivial, with only a few significant correlations falling within the small range. Comparatively, ACC was significantly correlated with the largest number of performance indicators, while REB was significantly associated with the greatest number of external load indicators. No significant differences in external load were found between players with better and worse performance. Overall, these findings provide only partial support for our hypotheses: while some small associations were observed, most relationships were weaker than expected, and external load did not distinguish between performance groups. These results may suggest that external load alone cannot accurately predict game performance, and that multidimensional factors and contextual conditions should be considered in evaluating players’ performance.

To our knowledge, this is the first study to evaluate the relationship between external load and game performance in 3 × 3 basketball. Therefore, comparisons can only be made with previous studies conducted in traditional 5 × 5 basketball. Current research distinguishes between training load and game load, with no clear relationship identified between training load and game performance. Specifically, Vázquez-Guerrero found that, in professional basketball, external training load was not related to Winscore, performance index rating, or player total contribution [26]. Two additional studies on semi-professional players reached similar conclusions, showing that higher training loads did not correspond to better game performance [23,46]. In contrast, studies on game load have reported divergent results across different populations. Studies on female and youth players have mostly reported associations between game load and game performance. One study in female collegiate basketball reported moderate-to-large correlations between game load and specific performance indicators, including points, field goals, and free-throw metrics [47]. Similarly, research on semi-professional female players reported moderate-to-large correlations between external load and basic technical statistics (steals, turnovers, points, shot attempts), with additional moderate associations observed in advanced statistics (number of possessions, points per possession, percentage of offensive rebounds) [48]. Furthermore, Piñar reported that players with a higher Performance Index Rating (PIR) accumulated greater loads during games [49]. For youth samples, a study on U19 male players also reported similar findings, identifying a moderate correlation between PlayerLoad and player index rating [25]. Studies on collegiate male players have likewise reported that increased game load was significantly associated with game statistics [50]. However, at the professional men’s level, García collected in-game external load indicators, including total distance, high-speed running, and number of accelerations, along with performance measures such as Performance Index Rating, player total contribution, and points scored, and found correlations between external load and performance indicators ranging from trivial to small [24]. The contrasting findings may be explained by differences in game structure and determining factors across competitive levels. Professional men’s basketball games exhibit greater intensity of physical confrontation and higher tactical complexity compared with semi-professional, collegiate, or female basketball. In elite-level competitive contexts, game performance tends to rely more heavily on players’ control of finer details, such as accuracy of tactical execution, quality of in-game decision-making, and technical skill execution [51], thereby limiting the explanatory power of external load.

Our findings are consistent with those reported in professional 5 × 5 basketball research [24]. Despite differences in court dimensions, player numbers, game characteristics, and rules, the absolute physical output data may differ between the two formats, and existing studies have confirmed differences in physical demands [1,3]. However, it should not be overlooked that both formats are high-intensity intermittent sports and share similar technical and tactical actions during competition [1]. Considering that the sample in this study was drawn from the Olympic Games, it effectively represents the highest competitive level and elite players in 3 × 3 basketball. Therefore, at the elite level, 3 × 3 and 5 × 5 basketball appear to exhibit similar relationships between physical output and game performance. In fact, available studies in 3 × 3 basketball suggest that external load differs only marginally between winning and losing games [14]. Similarly, Sansone found that although players reported higher perceived fatigue in lost games, average and peak heart rates showed little difference between wins and losses [2]. This may indicate that the physical and physiological demands imposed by the high intensity and fast pace of 3 × 3 basketball are experienced by every player on the court, while the smaller court size restricts players’ movement range, making these demands more uniform. Therefore, performance evaluation should adopt a holistic perspective, as external load may only reflect the degree of participation rather than serving as a decisive variable directly determining performance.

Among all external load indicators in this study, ACC showed significant correlations with the greatest number of performance metrics, including PTS, REB, 1PT made, KAS, and DRV. Nevertheless, it should be emphasized that these correlations remained within the small range, limiting their explanatory power. From a practical perspective, ACC may better reflect the high-intensity confrontations and explosive actions specific to 3 × 3 basketball compared with other external load indicators. As this sport heavily relies on neuromuscular actions (e.g., jumping, changes in direction, accelerations, and decelerations), previous studies have also emphasized the importance of instantaneous high-intensity actions and neuromuscular training in 3 × 3 basketball [1,2,6]. Due to the smaller court size and the absence of full-court transitions, sudden stops, starts, and changes in direction appear to play a relatively important role in half-court transitions and scoring opportunities. Meanwhile, REB emerged as the performance variable most correlated with external load indicators, including total distance and distances across different speed zones. This suggests that rebounding is not only related to players’ strength and physical contests but may also be associated with their overall movement coverage on the court. However, similar to ACC, these correlations remained small, implying that external load has limited explanatory power for rebounding performance. Overall, although ACC and REB demonstrated greater sensitivity than other indicators, they should be interpreted with caution in practice, as they are not strong predictive tools and cannot be used independently to assess game performance.

When examining the relationship between external load and performance, we found that external load could not distinguish between players with better or worse performance. This finding contrasts with research in traditional 5 × 5 basketball, where time-window analyses comparing elite and non-elite male players revealed that non-elite players experienced greater external demands, attributed to lower movement efficiency and differences in tactical adjustment capacity [10]. The discrepancy may be due to the characteristics of 3 × 3 basketball, in which every player on the court is required to take on comprehensive roles including playmaking, driving, and defending. Such role blurring and balanced task distribution further contribute to the convergence of players’ physical output patterns. In fact, when quantifying individual differences in external load, traditional 5 × 5 basketball often uses player position as a basis for classification. For example, available studies indicate that perimeter players exhibit significantly lower acceleration and deceleration rates compared with forwards and centers, while forwards display the lowest total external load in competition [52]. Conversely, in youth competitions, frontcourt players were found to sustain higher external loads than backcourt players [53]. Compared with other positions, centers showed statistical performance more closely related to the physical loads sustained in competition, which was explained by their reliance on physical displacement during offensive actions [54]. These findings provide useful references for designing position-specific training programs and contribute to more precise evaluations of player performance. However, such designs are difficult to implement in 3 × 3 basketball research, as players do not have clearly defined court positions. To further clarify the relationship between external load and performance in 3 × 3 basketball, future studies could consider grouping players based on functional roles, tactical–technical tasks, or clustering approaches, thereby providing a better explanation of external load contributions to performance.

Methodologically, this study is the first to apply a markerless motion capture system in real match settings of 3 × 3 basketball. Inertial systems are restricted by regulations in official international competitions, and traditional optoelectronic systems (e.g., Vicon) require lab environments and are expensive. In contrast, markerless motion capture is characterized by its non-invasive nature and operational flexibility, offering researchers and practitioners a feasible means of acquiring external load data during elite competitions like the Olympics. In particular, it has demonstrated strong accuracy and consistency in 3 × 3 basketball tasks [37]. This method is considered a practical solution for dynamic and complex team sport environments, with strong potential for broader implementation and scalability [55].

### Practical Applications, Limitations, and Future Directions

From a practical perspective, the findings of this study suggest that coaches and strength and conditioning practitioners should exercise caution when interpreting game performance using external load data. External load alone is insufficient as a determinant of performance quality and should instead be primarily applied to regulate training volume and monitor physical condition. The continuous monitoring of indicators such as PlayerLoad, running distance, and accelerations can assist coaches in appropriately distributing training and recovery cycles, thus preventing fatigue accumulation or overtraining from congested schedules. Moreover, integrating internal load indicators (e.g., heart rate, lactate, RPE) can more comprehensively reflect players’ physiological stress, offering more reliable references for pre-game preparation and in-game load management.

This study has several limitations. First, the sample included only female players from the Olympic Games. Although the sample represents the highest level of 3 × 3 basketball competition, the findings should be cautiously generalized to male players or other competitive levels. Second, no internal load data were collected. Multimodal data would provide deeper insights into the multidimensional determinants of player performance. Furthermore, the moderating effects of game context were not considered, although such factors have been widely applied in prior performance analyses. Future studies should aim to establish a more comprehensive monitoring framework. At the same time, given the specific scheduling characteristics of 3 × 3 basketball, exploring load and performance fluctuations under different contextual conditions may hold greater practical value.

## 5. Conclusions

This study is the first to use a markerless motion capture system to quantify external load in female 3 × 3 basketball players during the Olympic Games and to evaluate its relationship with game performance. The results provided no clear evidence of an association between external load and game performance, nor were significant differences in external load observed between players of differing performance levels. These results suggest that, in elite-level 3 × 3 basketball, external load primarily reflects participation and effort rather than serving as a key determinant of performance. This study provides new empirical evidence on the characteristics of 3 × 3 basketball, indicating that coaches and strength and conditioning practitioners should be cautious in using external load as a performance evaluation tool, as it may be more appropriate for training regulation and fatigue monitoring.

## Figures and Tables

**Table 1:** Game-related statistics used to represent player performance in 3 × 3 basketball.

Variable	Abbreviation	Definition
Points	PTS	No. of points scored by a team.
Rebounds	REB	No. of controlled recoveries of a live ball by a team being entitled to the ball for a throw-in after a missed shot or the last free-throw.
Highlight	HGL	A comprehensive evaluation variable; the calculation formula is KAS + DRV + DNK + BS + BZR
Shooting efficiency	S-EFF	The realized points-per-shot value for a shot. It is calculated by dividing the points made with the no. of shots attempted; the calculation formula is PTS/(1PTA + 2PTA + FTA)
1-point shots made	1PT made	No. of 1-point shots attempt for which 1 point was awarded.
1-point shots missed	1PT missed	No. of 1-point shots missed.
1-point shots attempt	1PT attempt	No. of 1-point shots attempted (both made and missed 1-point shots, excluding free-throws).
1-point %	1PT%	The shooting percentage for 1-point shots; the calculation formula is 1PT made/1PT attempt.
2-point shots made	2PT made	No. of 2-point shots attempt for which 2 points were awarded.
2-point shots missed	2PT missed	No. of 2-point shots missed.
2-point shots attempt	2PT attempt	No. of 2-point shots attempted (both made and missed 2-point shots, excluding free-throws).
2-point %	2PT%	The shooting percentage for 2-point shots; the calculation formula is 2PT made/2PT attempt.
Free-throws made	FT made	No. of free-throw shots attempt for which 1 point was awarded.
Free-throws missed	FT missed	No. of free-throw shots missed.
Free-throws attempt	FT attempt	No. of free-throw shots attempted (both made and missed free-throw shots).
Free-throw %	FT%	The shooting percentage for free-throw shots; the calculation formula is FT made/FT attempt
Key assists	KAS	No. of passes that give a teammate a positional advantage to score a basket directly from the paint.
Drives	DRV	No. of quick or skillful dribbling moves used to move from behind the two-point arc to directly score a field goal from the restricted area.
Dunks	DNK	No. of shots made in which the ball is forced by the player downwards into the basket with one or both hands.
Blocked shots	BS	No. of blocked shots that take place when a defending player contacts the ball to alter the flight of the shot attempt of the opponent, and the shot is missed.
Buzzer beaters	BZR	No. of last shots of the overtime, or tie-breaking, tie-forcing, or lead-changing last-made shots during the last 5 continuously played seconds of regular time (regardless of whether the game was won at the end of 10 min or before the limit, if a team reached 21 points).
Offense Rebounds	OREB	No. of rebounds awarded when the ball possession is retained by the same team who missed the field goal or the free throw.
Defense Rebounds	DREB	No. of rebounds awarded when the ball possession is gained by the other team who missed the field goal or the free throw.
Turnovers	TO	No. of mistakes committed by a team that results in the defensive team gaining possession of the ball.
Possessions	POS	No. of times a team had the ball and produced one of the following possession outcomes: 1-point shot, 2-point shot, trip to the free-throw line, or turnover.
Points per possession	PPP	No. of points a team scored per possession.
1-point possessions	POS-1PT	No. of times a team had the ball and attempted a 1-point shot.
1-point points per possession	PPP-1PT	No. of points a team scored per 1-point possession.
Free-throw possessions	POS-FT	No. of times a team had the ball and secured a trip to the free-throw line.
Free-throw points per possession	PPP-FT	No. of points a team scored per free-throw possession (excluding extra free-throw possessions).
2-point possessions	POS-2PT	No. of times a team had the ball and attempted a 2-point shot.
2-point points per possession	PPP-2PT	No. of points a team scored per 2-point possession.

**Table 2:** The difference in external load between better and worse performance.

External Load Metrics	Better Performance (*n* = 63)		Worse Performance (*n* = 128)		F	*p*
	Mean	SD	Mean	SD		
PlayerLoad	13.618	1.303	13.834	1.557	1.597	0.211
Total Distance	104.729	8.848	104.977	11.222	<0.001	0.979
LIA	62.926	6.242	62.495	8.886	0.660	0.418
MIA	36.369	5.360	36.868	8.930	0.254	0.615
HIA	5.435	2.362	5.615	3.543	1.002	0.318
ACC	4.989	2.125	4.888	2.857	0.139	0.709

## Data Availability

The raw data supporting the conclusions of this article will be made available by the authors on request.
